# Multi-Sensor Fusion Simultaneous Localization Mapping Based on Deep Reinforcement Learning and Multi-Model Adaptive Estimation

**DOI:** 10.3390/s24010048

**Published:** 2023-12-21

**Authors:** Ching-Chang Wong, Hsuan-Ming Feng, Kun-Lung Kuo

**Affiliations:** 1 Department of Electrical and Computer Engineering, Tamkang University, New Taipei City 25137, Taiwan; wong@ee.tku.edu.tw (C.-C.W.); lung40404@gmail.com (K.-L.K.); 2 Department of Computer Science and Information Engineering, National Quemoy University, Kinmen County 89250, Taiwan

**Keywords:** simultaneous localization and mapping (SLAM), deep reinforcement learning (DRL), multi-model adaptive estimation (MMAE), sensor fusion

## Abstract

In this study, we designed a multi-sensor fusion technique based on deep reinforcement learning (DRL) mechanisms and multi-model adaptive estimation (MMAE) for simultaneous localization and mapping (SLAM). The LiDAR-based point-to-line iterative closest point (PLICP) and RGB-D camera-based ORBSLAM2 methods were utilized to estimate the localization of mobile robots. The residual value anomaly detection was combined with the Proximal Policy Optimization (PPO)-based DRL model to accomplish the optimal adjustment of weights among different localization algorithms. Two kinds of indoor simulation environments were established by using the Gazebo simulator to validate the multi-model adaptive estimation localization performance, which is used in this paper. The experimental results of the proposed method in this study confirmed that it can effectively fuse the localization information from multiple sensors and enable mobile robots to obtain higher localization accuracy than the traditional PLICP and ORBSLAM2. It was also found that the proposed method increases the localization stability of mobile robots in complex environments.

## 1. Introduction

Manufacturing industries have been transforming their digital production into semi-automated or even automated operations in recent years, but many industries continued to wait and see until 2019 if the impact of the new coronavirus accelerated their transformation to automated smart factory production. Under this impact, the demand for automation-related products increased dramatically; however, Automated Guided Vehicles (AGVs), which play an important role in automation, can only travel along predefined paths, which is extremely inconvenient in terms of flexibility.

Light Detection and Ranging Simultaneous Localization and Mapping (LiDAR SLAM) and Visual SLAM based on cameras are the mainstream sensors used in simultaneous localization and mapping. LiDAR mainly uses a laser to measure the distance and other position information between targets. Lasers have higher accuracy in distance measurement, but their localization response is easily affected by the environment’s unclear texture. One of the popular studies in this field is LiDAR SLAM, such as GMapping [1] and Hector SLAM [2]. The camera mainly obtains the image or in-depth information through the imaging principle. However, the accuracy of its distance measurement is limited, and it is more likely to be affected by factors in the optical line environment. Similarly, the use of Visual SLAM such as ORBSLAM [3] and LSD-SLAM [4] has been studied widely. The environmental data obtained from a single sensor is limited and easily affected by factors such as sensor accuracy, algorithms, and the environment. Therefore, some studies added different sensors to assist the main sensor in obtaining better localization, such as SLAM-R with Radio Frequency Identification (RFID) [5] and ORBSLAM3 [6] with Inertial Measurement Unit (IMU). W. Wei et al. used 2D-3D hybrid dual-LiDAR to perform six DOF pose estimation and mapping tasks on a ground vehicle platform [7].

Nowadays, most of the commercially available mobile robots generate more information through different sensors to get better localization results. Most of the latest robot designs are equipped with both 2D LiDAR and a camera; usually, the 2D LiDAR is used for SLAM, and the camera is responsible for dynamic obstacle detection. One study [8] mentioned that LiDAR and cameras have better complementary characteristics and can integrate the advantages of LiDAR SLAM and Visual SLAM, which is a very popular and worthwhile research topic [9,10].

In the study of robot localization, it was found that the filtering algorithm has an absolute impact on the localization performance. In terms of information fusion, the Kalman Filter (KF) provided good results for linear approximation. Also, the extended Kalman Filter (EKF) and the Unscented Kalman Filter (UKF) provided good localization results for complex and non-linear features [11,12]. The multi-model adaptive estimation method (MMAE) has been known to play a very important role in multi-model adaptive control, mainly because it is highly suitable for predicting the values of uncertain parameters or variables [13,14]. The main logic of the MMAE method is that the sum of the likelihood function values of the unknown elements can be summed up by the weighting of the sub-filter’s estimation. Therefore, it can appropriately assess the degree of impact of each subtopic and strengthen the robustness of the overall system [15]. This is a very interesting research topic in the field of robotics in multi-sensor fusion.

Traditional MMAE is mainly based on a set of parallel KF, and the conditional probability of each hypothetical fault is given by a hypothesis-checking algorithm [16]. Conventional MMAE has several limitations, such as the limited number of filters, which limits the possible range of faults that can be covered. The limited detection range makes the residual output biased, and the accuracy of the state variable estimation is poor [17]. Thus, based on the above application constraints, for time-varying nonlinear aircraft control systems, it is important to understand how to combine UKF and MMAE with the robot fault diagnosis methodology of the hypothesis-checking algorithm. It is important to realize that the estimation of the probability of possible failures when the sensor systems are fused is an important element in multi-sensor fusion research [18]. Filter-based solutions using KF and their variants, such as EKF and UKF [19], can be quickly analyzed for their observability, which is a significant advantage [20]. Lynen et al. [21] proposed a loosely coupled multi-sensor fusion framework based on EKF for combining IMUs, GPS, and cameras. The framework can manage sensor data at different frequencies and assist in sensor interruptions. Meanwhile, Shen et al. [22] used a UKF-based odometry framework for cameras, radar, and GPS sensors. Many filter fusion techniques use global information and visual landmarks to update state estimates and achieve better performance.

Deep Reinforced Learning allows interactive autonomous learning and training for environments that cannot be known in advance. Rescue robots traveling over rough terrain use DRL and PPO to complete the planning of the climbing trajectory [23]. The reward function in DRL can guide mobile robots to simultaneously achieve safety, efficiency, and the goal of saving at least 60% of their energy [24]. PPO enhances the learning method that automatically adjusts the process noise covariance of the best-performing learning method [25] to achieve the target value of optimal tracking performance. It has a successful learning ability to effectively capture the appropriate reward under different environmental conditions, which in turn generates the optimal weighting values. Several papers have explored the use of PPO for behavioral parameter adjustment and localization of robots [26].

In order to increase the localization stability of mobile robots in complex environments with high accuracy, this paper proposes a multi-sensor localization method based on a two-dimensional LiDAR, an RGB-D camera, and a motor encoder. Moreover, we proposed a LiDAR SLAM and a Visual SLAM to adjust the weighted localization prediction of two different localization algorithms by PPO, allowing them to obtain a superior localization effect. The anomaly detection method in the localization algorithm was added to exclude the anomalous localization prediction condition to achieve a more robust localization effect.

## 2. Multi-Sensor Fusion-Based System Structure

**Figure 1:**
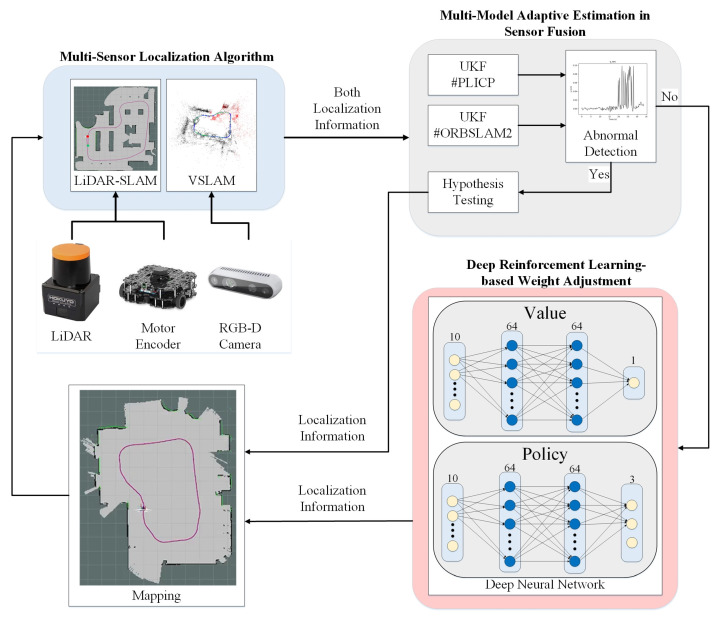
Architecture diagram of the proposed multi-sensor fusion-based simultaneous localization and mapping (SLAM) localization system.

The multi-sensor fusion architecture proposed in this paper is shown in Figure 1. An Intel Realsense D435i camera, a Hokuyo UTM-30LX optical radar, and a motor encoder were used as the sensors of the system to provide RGB image, depth, 2D point cloud, and motor odometry information about the environment. In multi-sensor localization algorithms, the output of 3-degree-of-freedom (*X*-axis displacement, *Y*-axis displacement, *Z*-axis rotation angle) localization is performed by Point-to-Line Iterative Closest Point (PLICP), and the output of 6-degree-of-freedom (*X*-axis displacement, *Y*-axis displacement, *Z*-axis rotation angle, Roll, Pitch, Yaw) is performed by ORBSLAM2. However, only 3-degree-of-freedom (*X*-axis displacement, *Y*-axis displacement, and *Z*-axis rotation angle) output by the ORBSLAM2 was used in this study. From the multi-model adaptive estimation in sensor fusion, this paper designed an algorithm to detect anomalies in the localization, predict the probability of anomalies through the UKF, and adjust its parameters for excluding the abnormal. The algorithmic process will be entered into deep reinforcement learning-based weight adjustment with no abnormalities. The DRL training machine is used to generate appropriate weight adjustments in different environmental situations. Finally, a 2D map is created by the occupancy grip map to complete the localization.

The multi-sensor fusion-based system proposed in this paper consists of three parts: (1) multi-sensor localization algorithm; (2) multi-model adaptive estimation in sensor fusion; and (3) deep reinforcement learning-based weight adjustment. The localization algorithms for the different sensors are introduced in multi-sensor localization algorithms. Meanwhile, in multi-model adaptive estimation in sensor fusion, the methods used for detecting anomalies and the mechanism for estimating the variability to be made after an anomaly occurs are introduced. Lastly, the architecture used in deep reinforcement learning, as well as the design concept of the reward function and timely weight adjustment, are introduced. Therefore, the localization prediction can be closer to the actual position and reduce the error introduced in the deep reinforcement learning-based weight adjustment.

### 2.1. Multi-Sensor Localization Algorithms

For the first step of the proposed localization algorithm in the concept of sensor fusion in this study, the sensor data from LiDAR, motor encoder, and RGB-D camera were utilized at the same time. The design of the multi-sensor localization algorithm was realized by LiDAR SLAM and Visual SLAM. The integration of the idea of selecting individual strengths was combined to establish better localization accuracy.

**Figure 2:**
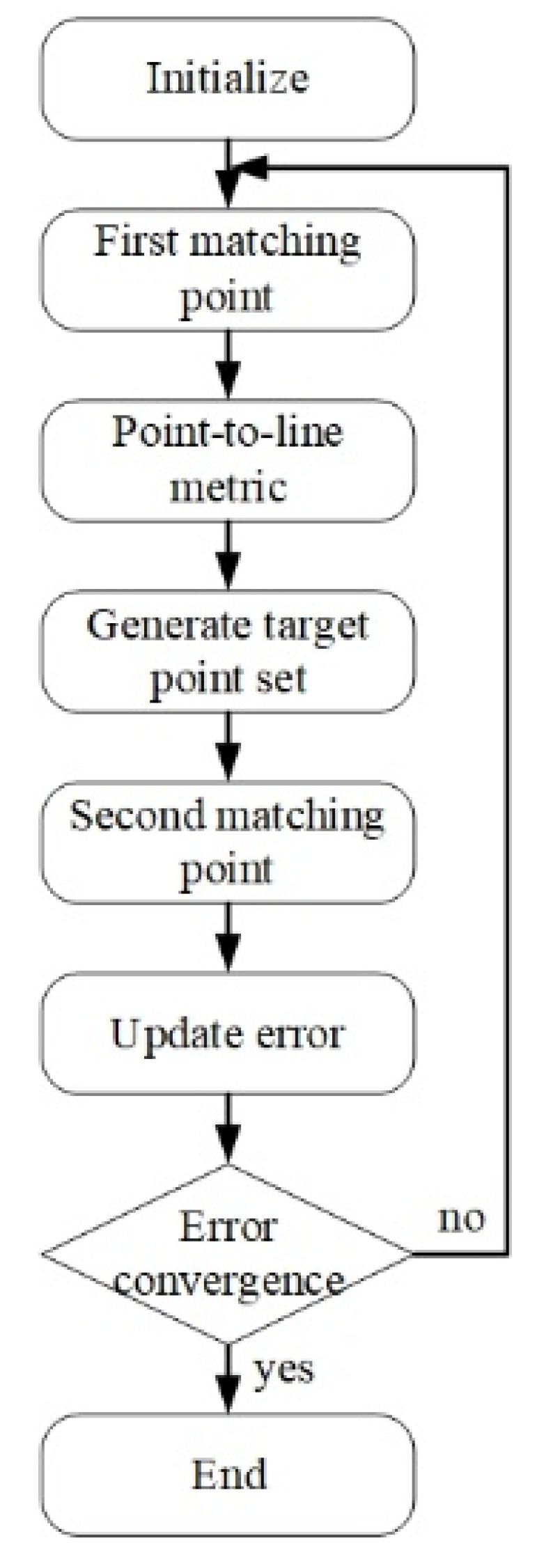
PLICP flow chart.

Firstly, since LiDAR has many advantages in distance measurements, such as fast computation speed and high accuracy, the LiDAR SLAM was used as the main advantageous factor in the design. The localization of SLAM is roughly divided into three types: (1) particle filter, (2) graph optimization, and (3) scan matching. Particle filters can obtain accurate localization by updating the weights of the particles. But over time, they consume more storage, resulting in a significant reduction in computation speed. The graph optimization can get a high-accuracy position through global, consistent optimization of attitude estimation and map construction. It needs to occupy larger computational resources for large environments, so the optimization time may be longer. Scanning matching can instantly estimate the position change and attitude change of the mobile robot. Through the comparison data between the previous frame and the current frame, the current position change can be deduced, resulting in higher accuracy. To achieve low computing costs and high precision, this paper selected the Iterative Closest Point (ICP), which is a point-to-point scanning matching method that meets the localization function needed [26]. The ICP method is mainly based on the pairing point relationship between the geometric data in the previous frame and the current frame. Using this pairing relationship and after many iterations, the displacement and rotation relationship of the converged two data is calculated, which belongs to the rigid transformation. In recent years, many researchers have developed improvements for the ICP method, such as the ICN [27], the weight ICP [28], etc. This study utilized a variant of the point-to-line approach called Point-to-Line Iterative Closest Point (PLICP) [29]; the flowchart of PLICP is shown in Figure 2. The main feature of this method is the speed and accuracy of the calculation. The first matching point step is aimed at building the point cloud of the previous frame and the current frame into a point collection, respectively. The next step is the first matching point collection, which is the value obtained after the first conversion relationship is calculated from the two points’ collections. The generated target point set is created by using a point-to-line metric and keeping 80% of points with the smallest displacement as the new target point set. The second matching point is calculated with the point cloud of the previous frame to obtain the second conversion relationship. Finally, the update error is calculated in the previous frame and the current frame of the error value, and the error convergence threshold is used for comparison to determine whether to end the iteration. If the continuous smoothness of the point clouds on both sides is too high, it is easy to reach the convergence threshold in advance, which could lead to errors in the minimization of the generated area. In order to solve this problem, this study increased the amount of variation predicted by the motor odometry, which is input simultaneously with LiDAR and used as the initial conversion of PLICP. This method can effectively solve the problem of area minimization and reduce the number of iterations.

This paper used the displacement variation and rotation variation calculated by the odometer information from motor encode to act as the first data conversion. The effective distance parameter of the corresponding point was 0.5 m. The receiver initially finds the nearest point of each target point $b_{j}$ in the set *A*, through the rotation angle $\hat{\theta }$, the rotation matrix $\hat{R},$ and the minimum translation matrix $\hat{T}$; then, the conversion relation is calculated. The displacement change and rotation change calculated by the odometer information were utilized as the first data conversion. Through Equation (1), the set of transformation target points $B^{\prime }=\{b_{j}^{\prime },j=1,2,\ldots ,n_{sens}\}$ after the first transformation was obtained.

(1) $$b_{j}^{\prime }=\hat{R}(\hat{\theta })b_{j}+\hat{T}$$

The reference point set $A=\left\{a_{i},i=1,2,\ldots ,n_{ref}\right\}$ is the data of the alignment model, $a_{i}$ is the data points in set *A*, and $n_{ref}$ is the number of data in set *A*; while the target point set $B=\left\{b_{j},j=1,2,\ldots ,n_{sens}\right\}$ is the data that needs to be aligned, $b_{j}$ is the data points in set *B*, and $n_{sens}$ is the number of data in set *B*, in which the number of $n_{ref}$ and $n_{sens}$ may not necessarily be the same.

In the selection of Visual SLAM, this paper used ORBSLAM2, which has an outstanding achievement in accuracy [30]. The localization part mainly uses the conversion method from one feature point to another feature point in scanning matching. The final optimization step is carried out through the graph optimization method. The use of Oriented Fast and Rotated Brief (ORB) feature points in the proposed ORBSLAM2 method has a great advantage in terms of computational speed compared to other different feature point algorithms. Also, the feature points have the properties of scale invariance and rotational invariance. To facilitate the application of mobile robots in real-world scenarios, this paper reduced the resolution of the image and depth information from 1280 × 720 to 640 × 480, which effectively decreased the computational load on the robot’s movement. However, it affected the accuracy of localization. To obtain a better localization function, a small number of feature points and a larger number of keyframes were used. Since a decrease in the number of feature points leads to easier mismatching, increasing the distinguishability of the feature points can be used to increase the quality of each feature point at the same time. The Features from Accelerated Segment Test (FAST) algorithm [31] is a highly efficient feature detection method that can be updated almost in real time by recognizing corners as features. This was employed in this paper to increase the initial threshold of FAST to build up better feature points and decrease the minimum threshold. This was done to avoid getting lost because of the lack of feature points. Then, the number of Gaussian pyramid layers was increased to get better scale invariance for better localization results.

### 2.2. Multi-Model Adaptive Estimation in Sensor Fusion

In order to make the sensor-fused localization information more effective, anomalous localization information of LiDAR SLAM and Visual SLAM was detected and excluded to enhance the overall localization capability. This study referred to the framework of MMAE [32], which was originally applied to detect the anomalies of each sensor on an aircraft. The core idea is to filter the information from multiple sensors into their respective KFs and treat each KF as an independent model; then, the residual values calculated from each model are input into hypothesis testing to assess the probability of an anomaly. It provides an inventory of the overall anomalous condition. A more adaptive MMAE in the sensor fusion framework is proposed in this study, consisting of three parts: (1) UKF, (2) hypothesis checking, and (3) MMAE. These are described in detail below.

#### 2.2.1. Unscented Kamman Filter (UKF)

An important study [33] noted that when a KF model is perfectly matched to a real system model, the residuals of the filter are presented as a Gaussian distribution with a zero mean and known covariance. The basic assumption of the KF is that the system model is linear with Gaussian noise characteristics and follows the Gaussian distribution. The optimality of the filter chosen in the design of the prediction model proposed in this paper has a large impact on the performance and is an important step in the complete matching of the real system model. Since the position information of mobile robots is non-linear, the KF can only process linear data. This paper builds a filter that can process non-linear systems. The EKF is one of the improved variants of KF that is capable of handling nonlinear data. However, when it encounters strong nonlinear data, the accuracy is seriously reduced, prompting the need to calculate the Jacobian matrix of the nonlinear functions, which is not only more complex but is also prone to cause numerical instability and computational dispersion problems. The UKF [34] is another variant of the KF that is also capable of handling nonlinear systems and maintaining the Gaussian distribution. In terms of computational speed, it does not require the system to be linearized, which effectively reduces the computational burden. It is also easy to implement in real-time systems, which is in line with the requirements of the design of this paper. The core concept of the UKF is to transform the state estimation problem into a linear problem using Unscented Transform (UT). Therefore, the nonlinear system maintains the form of a Gaussian distribution in the state space, simplifying the number of calculations in the filter. By calculating a set of sample points called Sigma points, these sample points, after passing through the nonlinear system model, are then used to estimate the state of the prediction model through a weighted linear combination. The weights of the Sigma points are appropriately adjusted to minimize the error in the state estimation. Continuously iteratively updating the weights through the state observation values and the dynamic equations of the system improves the accuracy and stability of the overall estimation. The design of the UKF is described as follows:

Firstly, a similar KF needs to establish the state $X_{k}$ and observation $Z_{k}$, which are represented by Equations (2) and (3), respectively:

(2) $$X_{k}=f(X_{k-1},W_{k-1})$$

(3) $$Z_{k}=h(X_{k-1},\;V_{k-1})$$

where:

$W_{k-1}$ is the skewness of the last moment of state noise.
$V_{k-1}$ is the skewness of the last moment of observed noise.

After building the model, the two adjustment stages of prediction and correction are provided. For the prediction stage, the 2n + 1 sigma points, the set of sigma points $\varepsilon_{i}$, and the weights *ω* obtained from the traceless transformation are substituted into the state model to obtain the state mean X and the covariance $P_{x}$, which are calculated by Equations (4) and (5), respectively.

(4) $${\bar{X}}_{k|k-1}=\sum_{i=0}^{2n}\omega_{i}^{\left(m\right)}\varepsilon_{i,k|k-1}$$

(5) $$P_{k|k-1}=\sum_{i=0}^{2n}\omega_{i}^{\left(c\right)}[\varepsilon_{i,k|k-1}-{\bar{X}}_{k|k-1}]{\left[\varepsilon_{i,k|k-1}-{\bar{X}}_{k|k-1}\right]}^{T}$$

where:

${\bar{X}}_{k|k-1}$ is the state mean value;
$\omega_{i}^{\left(m\right)}$ is the weight of the mean of the *i*th Sigma point;
$\omega_{i}^{\left(c\right)}$ is the weight of the covariance of the *i*th Sigma point;
$\varepsilon_{i,k|k-1}$ is the set of predicted Sigma states.

Substituting the data obtained from the traceless transformation into Equation (2) yields the observation model $Z_{k}$ and the average value of the observation model ${\bar{Z}}_{k|k-1}$, which are represented by Equations (6) and (7), respectively:

(6) $${\bar{X}}_{k|k-1}=\sum_{i=0}^{2n}\omega_{i}^{\left(m\right)}\varepsilon_{i,k|k-1}$$

(7) $$P_{k|k-1}=\sum_{i=0}^{2n}\omega_{i}^{\left(c\right)}[\varepsilon_{i,k|k-1}-{\bar{X}}_{k|k-1}]{\left[\varepsilon_{i,k|k-1}-{\bar{X}}_{k|k-1}\right]}^{T}$$

For the correction stage, it is represented by Equations (8)–(12), respectively.

(8) $$P_{k|k-1}^{ZZ}=\sum_{i=0}^{2n}\omega_{i}^{\left(c\right)}[Z_{k|k-1}^{i}-{\bar{Z}}_{k|k-1}]{\left[Z_{k|k-1}^{i}-{\bar{Z}}_{k|k-1}\right]}^{T}$$

(9) $$P_{k|k-1}^{XZ}=\sum_{i=0}^{2n}\omega_{i}^{\left(c\right)}[\varepsilon_{i,k|k-1}-{\bar{X}}_{k|k-1}]{\left[Z_{k|k-1}^{i}-{\bar{Z}}_{k|k-1}\right]}^{T}$$

(10) $$K_{k}=P_{k|k-1}^{ZZ}{\left(P_{k|k-1}^{XZ}\right)}^{-1}$$

(11) $$X_{k}={\bar{X}}_{k|k-1}+K_{k}(Z_{k}-{\bar{Z}}_{k|k-1})$$

(12) $$P_{k}=P_{k|k-1}+K_{k}P_{k|k-1}^{ZZ}{K_{k}}^{T}$$

where:

$P_{k|k-1}^{ZZ}$ is the covariance of the state values from time *k* − 1 to time *k;*
$P_{k|k-1}^{XZ}$ is the covariance of the state values from time *k* − 1 to time *k* over the observations;
$K_{k}$ is the KF gain coefficient;
$Z_{k}-{\bar{Z}}_{k|k-1}$ is the residual value of the model.

**Figure 3:**
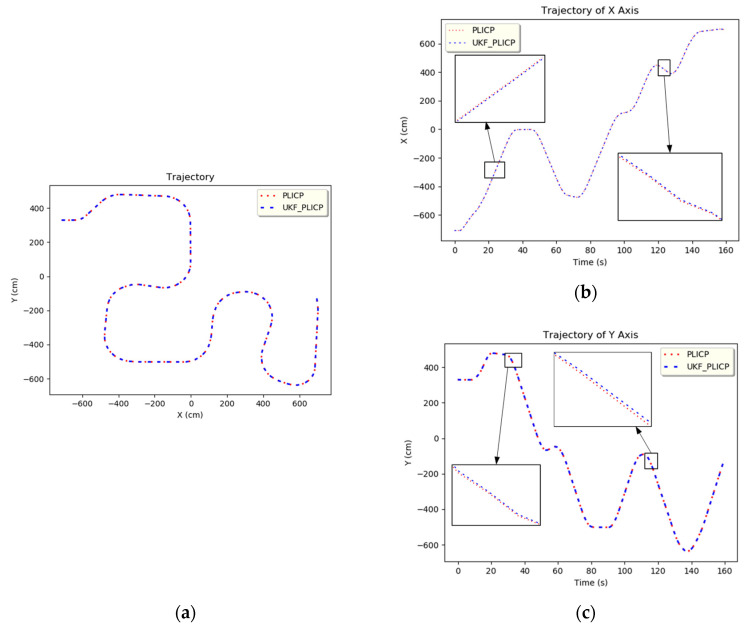
UKF trajectory of PLICP. (**a**) Full trajectory. (**b**) *x*-axis trajectory. (**c**) *y*-axis trajectory.

The localization information of PLICP was imported into the UKF, as shown in Figure 3. Figure 3a–c presents the overall trajectory plot, the *x*-axis trajectory plot, and the *y*-axis trajectory plot, respectively. The blue lines represent the original PLICP plots, and the red dotted lines are the PLICP plots after the UKF prediction. The best mean square error with PLICP was 0.0009 (cm) after the calculation.

**Figure 4:**
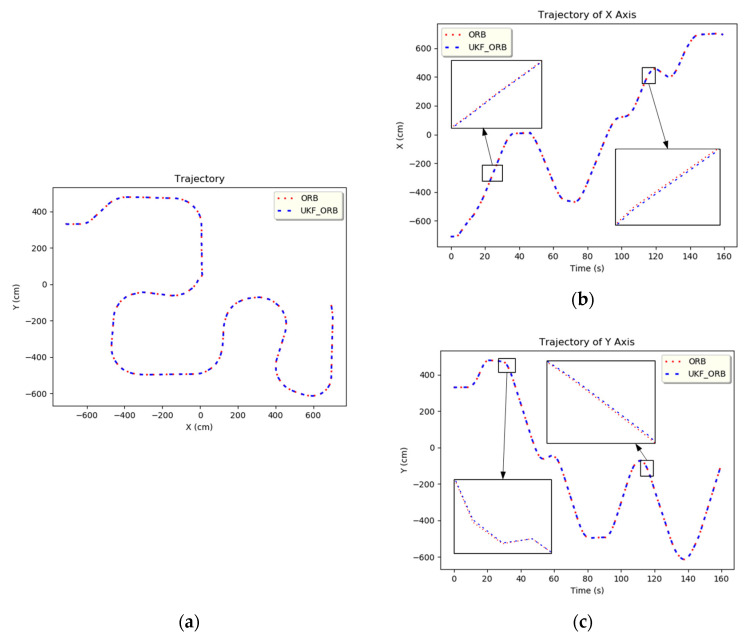
UKF trajectory of ORBSLAM2. (**a**) Full trajectory. (**b**) *x*-axis trajectory. (**c**) *y*-axis trajectory.

The ORBSLAM2 information was imported into the UKF as shown in Figure 4; Figure 4a–c shows the overall trajectory, the *x*-axis trajectory, and the *y*-axis trajectory, respectively. The blue line represents the original ORBSLAM2 image, and the red dotted line is the UKF-predicted ORBSLAM2 plot, which was calculated as the best mean square error of 0.0008 (cm) with respect to ORBSLAM2. The image data demonstrates that the UKF is indeed able to approximate the real system model to an almost perfect match. This proves that it can effectively predict the estimation of the localization algorithm. Therefore, its residual value can be used as a basis for determining system stability. Therefore, the extracted data from both PLICP and ORBSLAM2 serve as the basis for the information flow in the multi-sensor fusion-based model in this paper.

#### 2.2.2. Hypothesis Testing

Hypothesis testing is a statistical method for testing hypotheses based on sample data and determining whether the sample data supports or refutes a hypothesis through systematic judgment. This method is mainly used to determine whether the observations in the parent sample support a certain hypothesis. The null hypothesis and its alternative hypothesis, denoted as $H_{0}$ and $H_{1}$ respectively, are first set for the parent sample. Then, a comparison statistic and a critical value are used to determine whether the null hypothesis is rejected or not. Table 1 shows the hypothesis determination and hypothesis checking of the current study. If the statistic is greater than the critical value, the null hypothesis is rejected and the opposing hypothesis is accepted. Conversely, the null hypothesis cannot be rejected, and the opposing hypothesis cannot be proved.

The hypothesis testing decision contains type I error and type II error, which are two forms of misjudgment. Type I errors occur mainly when the virtual hypothesis is established. But the calculated testing statistics fall into the rejection area, rejecting the hypothesis. Type II error occurs when the false hypothesis is not valid but the calculated calibration statistic does not fall in the rejection region. It accepts the false hypothesis.

**Figure 5:**
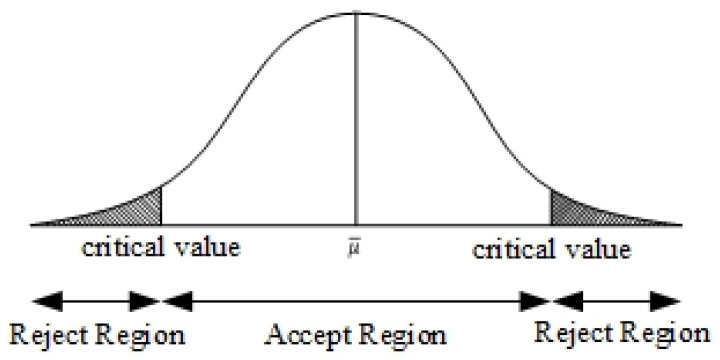
Two-Tailed validation.

In this paper, since the positive and negative directions of the changes in the error checking need to be considered and realized at the same time, the two-tailed Z-test was utilized as the main basis for judgment. The input of this system was the position information of the mobile robot as the standard. We established the residual value calculated by the UKF model so that when the position of the mobile robot was recognized as the standard of the hypothesis test, all the possible data of different directionality became the standard. The residual value (including positive and negative directions) was confirmed by the hypothesis test. A schematic diagram of the two-tailed validation is shown in Figure 5, wherein the standard deviation of the parent samples is assumed as $s_{h}$, the number of samples as $n_{h}$, and the mean of samples as ${\bar{a}}_{h}$.

The *z*-value in the Z-determination can be expressed as in Equation (13) below.

(13) z=a¯h−μ¯shnh

The *z*-value is used to evaluate the discrepancy between the sample results and the hypothesis. It is employed to determine whether there is any variation so that subsequent adaptive adjustments can be made.

#### 2.2.3. Multi-Model Adaptive Estimates

**Figure 6:**
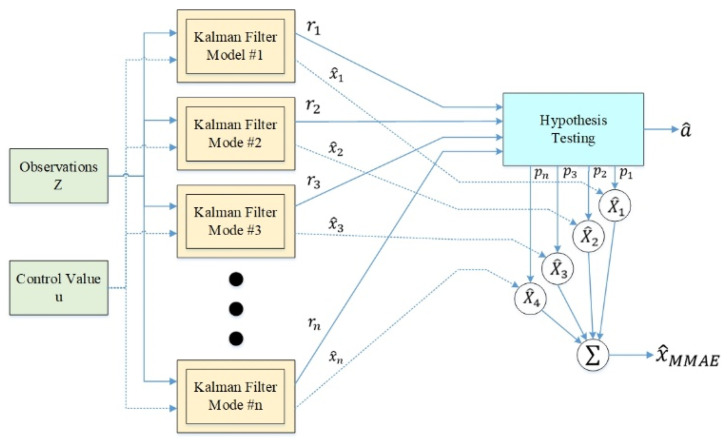
Original multi-model estimation schematic.

In this paper, we propose an improved MMAE design under the basic framework of the original multi-model estimation. Figure 6 presents the framework of the original multi-model estimation, which is mainly divided into two main parts, namely, model and hypothesis checking. In the model part, the observed *z*-value and the control quantity u of the sensor information were used as the inputs of the whole system, and the data of n sensors were put into their respective KF as different models. The residual value *r* was computed from the difference between the observed *z*-value and the state estimation $\hat{x}$ of each sensor, and the residual value of each model was used as the input to the hypothesis checking to estimate the true parameter vector $\hat{a}$ and to generate the appropriate posterior condition probability *p*. The posterior condition probabilities can be expressed as the relative correctness of each model, which is used to select the best estimator in a real system fault state. Finally, a suitable weighting operation of the state estimates through the posterior conditional probabilities led to the derivation of the probability-weighted average state estimate ${\hat{X}}_{MMAE}$. The model in the original multi-model estimation architecture design used a KF; however, the linear estimation characteristics of this model cannot reach a perfect match with the real system model. This was improved in this paper by choosing the UKF, which can handle the non-linear system. In addition, in the original multi-model adaptive estimation framework, the overall failure probability is mainly obtained by weighting the linear sum of individual failure probabilities and sensed values. In order to cope with the nonlinear characteristics of mobile robots when walking, this paper was designed to promptly detect the oscillations of the current multi-localization algorithms that may occur in the asynchronous state to avoid excessive errors from the mobile robots.

**Figure 7:**
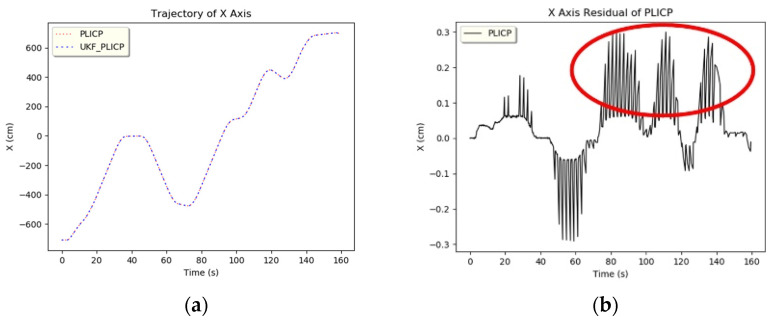
Mobile robot oscillates when turning a corner. (**a**) *x*-axis trajectory of PLICP. (**b**) *x*-axis residual of PLICP. (Red circle refer to the turning part).

**Figure 8:**
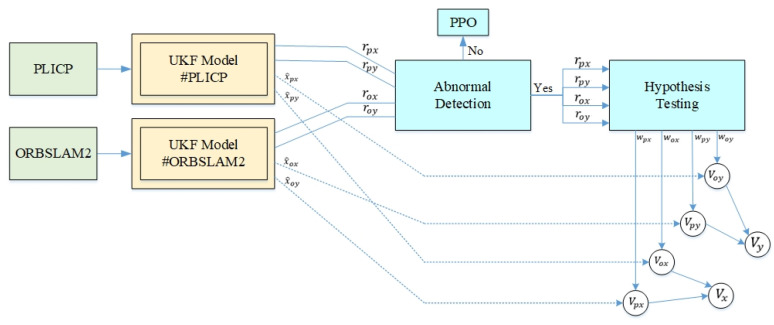
Multi-model adaptive estimation architecture.

The model used in the original multi-model estimation was the KF, and the expected input was the multi-sensory localization information of PLICP and ORBSLAM2. The result after inputting this localization information into the UKF is shown in Figure 7. It can be seen that when the mobile robot turned and moved, there was a change in acceleration and deceleration, which easily caused a large oscillation in the residual value. Usually, this kind of oscillation can lead to the misjudgment of hypothesis-checking. To solve this anomaly information error, this study added an input value based on the difference between the residual values of the two models; this was entered into the anomaly detection program. The improved model is called MMAE, and its structure is shown in Figure 8. In this paper, anomaly detection uses the difference between the residual values of the PLICP and ORBSLAM2 models as the basis for determining whether an anomaly occurs. That is, it is regarded as a condition to detect whether the two models in the current system are unstable or not. If the residual values of the two models oscillate at the same time, it is viewed as the mobile robot turning and filtering out the abnormal oscillations, which is a normal situation at this time. If the difference between the residual values of the two models is greater than the threshold value set for anomaly detection, it is considered one of the models having an anomaly. Thus, it is necessary to enter the hypothesis detection mechanism to detect the localization algorithm where the anomaly occurs.

For the threshold design, the current camera frame rate was set at 30 fps and the LiDAR frame rate at 5 fps. Assuming that the current mobile robot speed is $V_{m}$ m/s, to make the threshold more accurate in detecting abnormal events, the threshold $T_{m}$ was designed as in Equation (14):

(14) $$T_{m}=V_{m}/5$$

**Figure 9:**
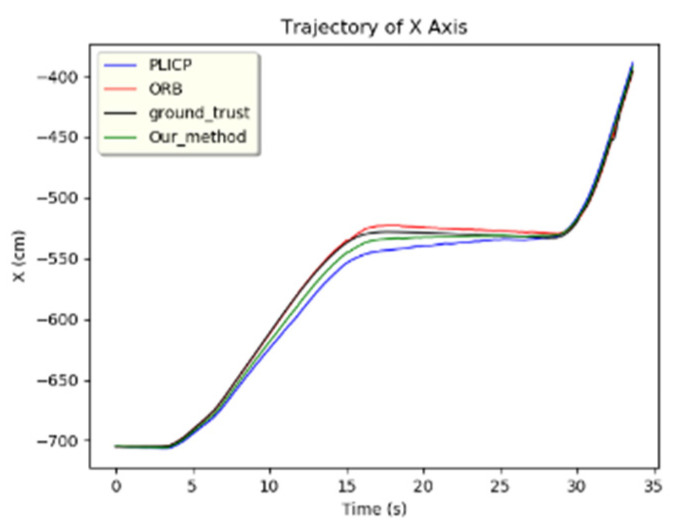
*x*-axis trajectory.

**Figure 10:**
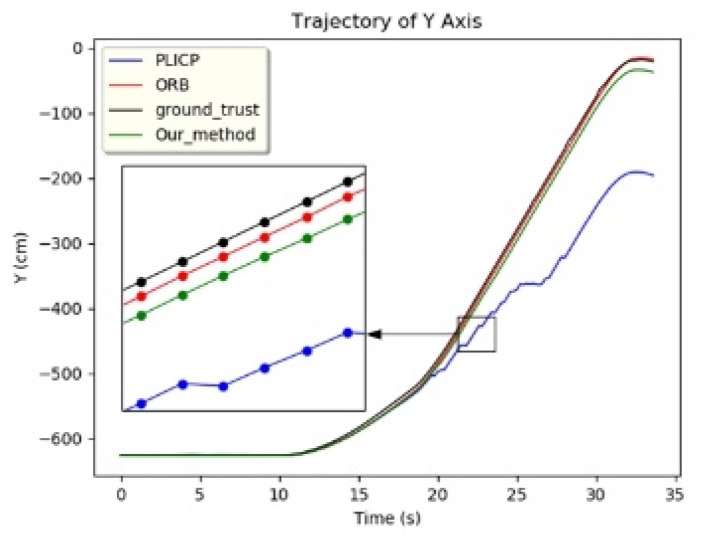
*y*-axis trajectory.

**Figure 11:**
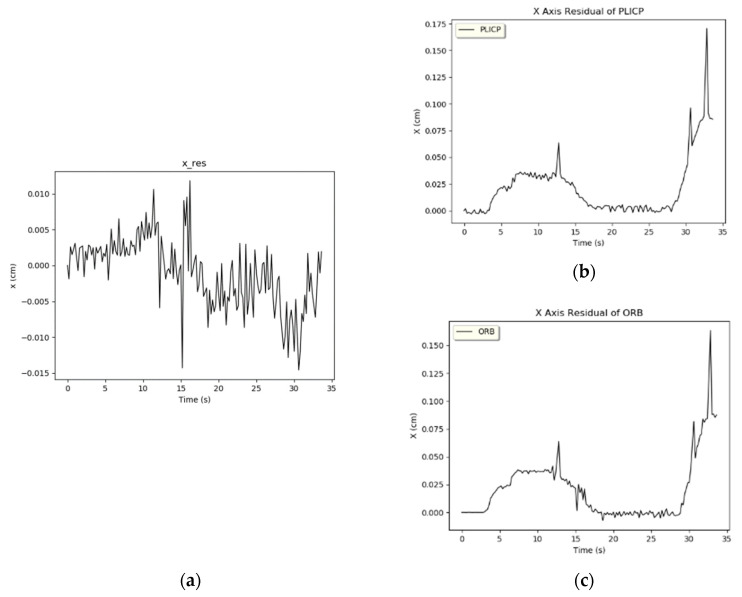
*x*-axis residual value. (**a**) *x*-axis difference. (**b**) *x*-axis residual of PLICP. (**c**) *x*-axis residual of ORBSLAM2.

When the difference between the residual values of the two models is larger than the displacement change of the current speed, it means that a model failure has occurred. The system enters into hypothesis-checking to carry out the detection program. During hypothesis-checking, the *z*-values of the *x* and *y* axes of the two models in the Z-test are calculated first. Since the *z*-value is used to evaluate the difference between the sample results and the hypothesis, a larger *z*-value means that this data has a greater degree of dispersion from the mean value of the parent sample, which is an anomaly. On the other hand, a smaller *z*-value means that the deviation of this data from the mean value of the parent sample is smaller, and the data is more stable. Through the judgment of the *z*-value, we were able to detect the current fault model and set the weight of this localization algorithm to 0. Then, it is combined with the state estimation value x of the UKF as the optimal variation of the mobile robot in this frame. Take the PLICP minimization fault event as an example, where the trajectories of the x and y axes are shown in Figure 9 and Figure 10, respectively. In the *x*-axis trajectory of Figure 9, it can be seen that there is no abnormality in the two localization algorithms. However, from the residual values of the *x*-axis in Figure 11b,c, it can be found that the residual values of the two models increased rapidly due to bending, and two large oscillations at about 30 s were observed. In Figure 11a, it can be noted that the difference in the residual values of the whole *x*-axis was much smaller than the current threshold value (set to 0.05). According to the specification of the anomaly detection design, it can be concluded that there was no anomaly in the position of the *x*-axis in the two algorithms.

**Figure 12:**
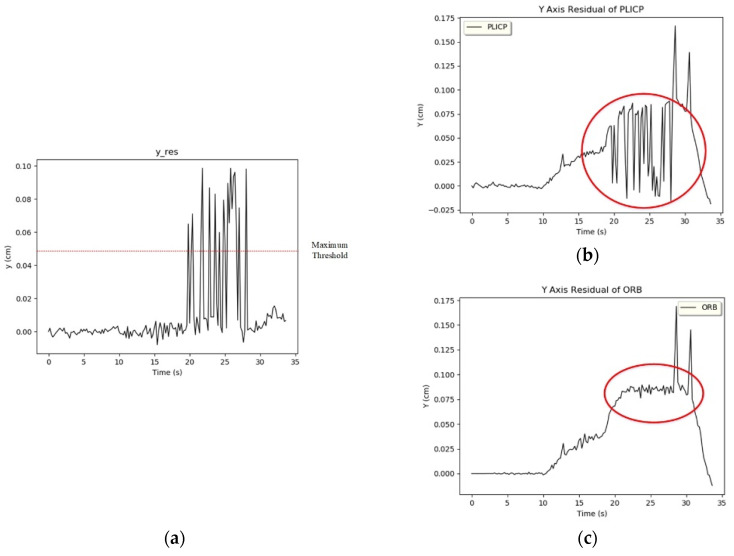
*y*-axis residual value. (**a**) *y*-axis difference. (**b**) *y*-axis residual of PLICP. (Red circle refer to the unstable response) (**c**) *y*-axis residual of ORBSLAM2. (Red circle refer to the stable response).

In the *y*-axis trajectory of Figure 10, it can be found that PLICP had an abnormal trajectory after 20 s. In Figure 12a, it can be seen that the difference between the *y*-axis residual values of the two models was larger than the threshold value (0.05) several times in the interval from 20 s to 30 s. In Figure 12b,c, we can see that ORBSLAM2 maintained a stable oscillation with a small amplitude after 20 s; on the contrary, PLICP had a large oscillation after 20 s. The difference between the residual values of the *y*-axis of the two models was estimated, and the result exceeded the threshold value. Thus, a hypothetical check fault was detected, and the weights were adapted to eliminate the occurrence of faults and increase the overall stability of the localization.

**Figure 13:**
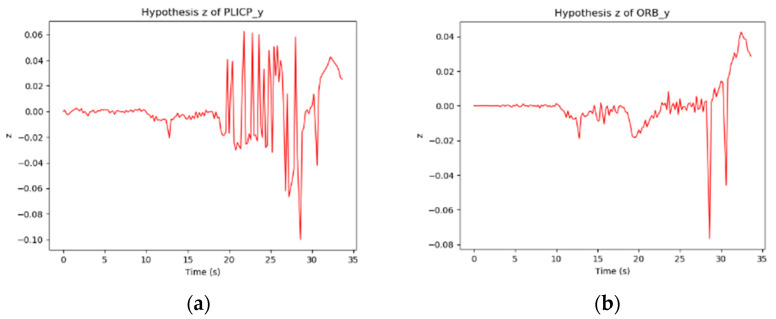
Mobile robot oscillates when turning a corner. (**a**) *z*-value of PLICP. (**b**) *z*-value of ORBSLAM2.

From Figure 13a, it is illustrated that the difference between the PLICP sample and the parent sample between 20 and 30 s can indeed be too large. It can be observed from the red circles of Figure 12b,c. On the contrary, there are only two larger oscillations in Figure 13b, but these two oscillations, as observed in Figure 12a, do not reach the threshold criterion. This phenomenon will be judged as simultaneous oscillations in the two models. Therefore, these two oscillations of ORBSLAM2 do not enter into the hypothesis-checking test.

**Figure 14:**
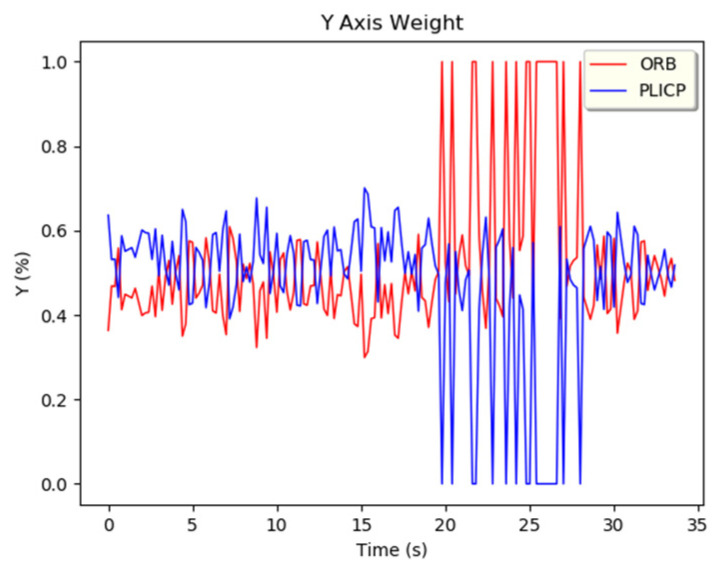
y-axis weight adjustment.

**Figure 15:**
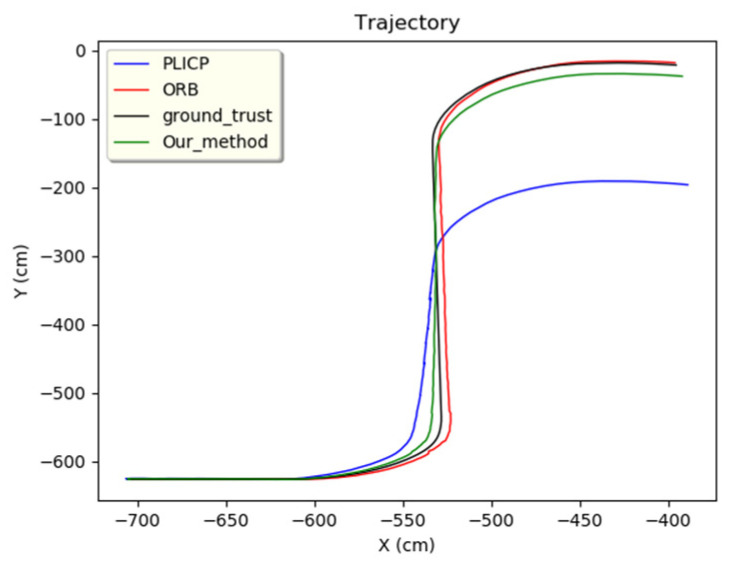
Overall trajectory of PLICP failure events.

For events larger than the threshold detection, the *z*-value of the sampled data is examined after entering the hypothesis check. When the *z*-value is larger, it means that the localization prediction of the model is more unstable. The estimation of the localization change is defined as a failure, and the weight is to be adjusted to 0 after setting. When the *z*-value is smaller, it means that the localization prediction of the model is more stable, so the estimation of localization variation is defined as successful, and its weight is adjusted to 1 after setting. From the experiments shown in Figure 14, it can be observed that in the region where PLICP had an anomaly (between 20 and 28 s), it was effectively detected and ruled out. In Figure 15, it can be seen that these anomalies generated by PLICP (blue line) did not affect the correctness of the trajectory prediction of the MMAE proposed in this study (green line).

### 2.3. Deep Reinforcement Learning-Based Weight Adjustment

In order to make the fused localization information more precise, this study proposed adaptive deep reinforcement learning (DRL) that can adjust the weights according to different environmental information. The robot learns and adapts the strategies to different scenarios and tasks in advance. This allows efficient motion planning, operation, and control of the robot.

**Figure 16:**
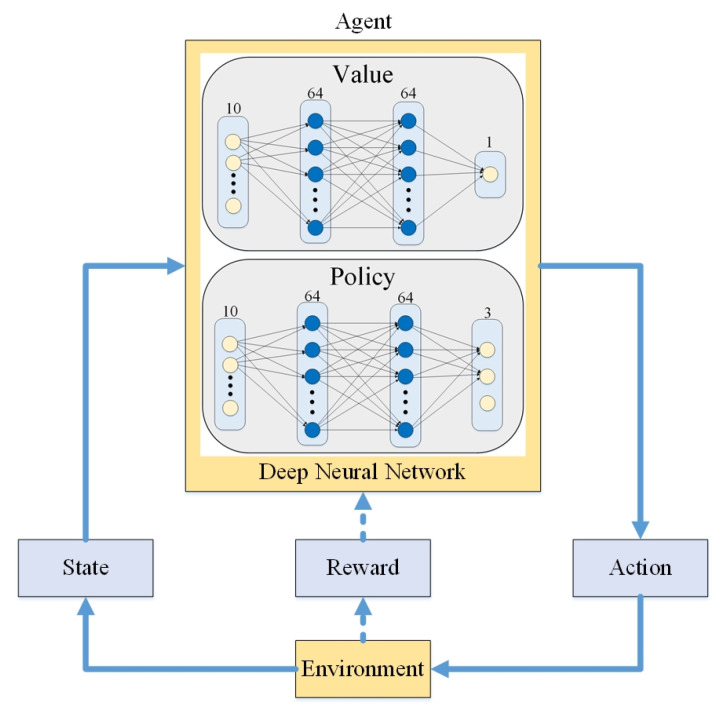
DRL structure.

Among them, Proximal Policy Optimization (PPO) [35] is a learning method based on policy optimization. PPO has the advantages of fast convergence, better stability, and an improved ability to solve high-dimensional or more complex problems. The architecture of PPO is shown in Figure 16. The core concept is to learn how to choose the appropriate action to get the maximum reward (reward) through the interaction between the agent and the environment based on the pretrained Deep Neural Network (DNN). Since the input of the DNN is the only part of the network that interacts with the environment, it is very important for the learning and performance of the network to give effective information appropriately. The DNN needs to provide effective information to the network to effectively adjust the weight of the current localization variations. Therefore, the adjusted localization variations after the weighted calculation can be more in line with the actual localization target of the mobile robot. In this study, the proposed two sensor localization algorithms were used as inputs to the DNN. However, having only two localization variations cannot effectively achieve optimal localization through training and learning of the external environmental inputs. Thus, the residual value, which can show whether the two model systems are stable or not, was used as a common input to the DNN. Therefore, the input information consisted of the ORBSLAM2 attitude change, the PLICP attitude change, the UKF residual value of ORBSLAM2, the UKF residual value of PLICP, and so on. Our goal was to estimate the position variations of mobile robots to meet the actual localizations, so the weight adjustment mechanism of the pretrained DNN is applied to satisfy the output targets of three variations, such as *x*-axis variations, *y*-axis variations, and *z*-axis rotation variations, to meet the actual variations.

In the reinforcement learning approach, the ultimate goal of the agent is to obtain the highest reward value; therefore, the behaviors generated by the designed agent and the reward value can establish a strong correlation. Therefore, the proportion of reward value reflected by good or bad behaviors should have the ability to adapt to changes in external environmental conditions. An ill-conceived reward function may end up with a good reward value, but it will mostly cause the agent to choose undesired behavior that leads to failure. On the other side of the training expectation, the agent may choose to sacrifice some points to quickly get better reward values. To guide the neural network to train the mobile robot with the correct amount of localization variation so that it can more closely match the real-time variation of the actual environment. This paper utilizes the concept of intensive reward design. This allows the required reward value to be given and adjusted according to the size of the ratio of the difference with the actual variation of the mobile robot. If it is smaller than the threshold value, the action meets the expectation and is rewarded positively. On the other hand, if it is greater than the threshold value, the action does not meet the expectation and is rewarded negatively. Since the two localization algorithms mentioned in this study are affected by different factors such as the accuracy of the sensors, the calculation of the algorithms, or external noise, the error between the final position change and the actual change may be too large.

To correct this error, we based the study on (1) the actual amount of variation between the ORBSLAM2 and PLICP localization algorithms of the localization change or (2) the actual amount of variation at the same time that is either greater than or smaller than the two localization algorithms of the localization variation. Through the analysis of these two different error ranges, this paper designed the intensive rewards (intensive rewards) with two types of correction: (1) variation localized in the interval and (2) variation outside the interval. Both are described in terms of (1) the amount of variation localized in the interval and (2) the amount of variation localized in the interval.

#### 2.3.1. The Amount of Variation Localized in the Interval

This situation applies when the actual change falls in the middle of the predicted variation of the two localization algorithms, i.e., if the conditions of Equation (15) or (16) are met, then it is categorized as a case within the variation interval.

(15) $$V_{Tru}\leq \;V_{ORB}\;and\;V_{Tru}\geq V_{PLICP}$$

(16) $$V_{Tru}\geq V_{ORB}\;and\;V_{Tru}\leq V_{PLICP}$$

where:

$V_{ORB}$ is the change of ORBSLAM2 at time *t;*
$V_{Tru}$ is the actual change of mobile robot at time *t;*
$V_{PLICP}$ is the change of PLICP at time *t.*

In the rewarding section, the difference between the variations of ORBSLAM2 and PLICP, $D_{max}$ is calculated, and one-fourth of the variation $D_{qua}$ is taken as the criterion for judgment. Later, the difference between the localized variations that are more different from the actual variations $D_{far}$, and the difference between the localized variations that are closer to the actual variations $D_{clo}$, can be calculated. If it conforms to Equation (17), it means that $V_{Tru}$ is within the center area of $V_{ORB}$ and $V_{PLICP}$.

(17) $$\left|V_{Tru}-V_{ORB}\right|\geq D_{qua}and\left|V_{Tru}-V_{PLICP}\right|\geq D_{qua}$$

For the positive reward value component, it is assumed that Equation (17) is satisfied, which means that this prediction is extremely close to the actual position of the mobile robot. Therefore, the reward value *R* is given based on the proportion of the distance difference between $V_{pre}$ and $V_{Tru}$, as shown in Equation (18). If Equation (19) is not satisfied, it means that the difference between $V_{pre}$ and the actual change is too large. Thus, a negative reward value is given, and this reward value R is adjusted according to the distance between $V_{pre}$ and $V_{Tru}$, as shown in Equation (20):

(18) $$R=1-\left|V_{pre}-V_{Tru}\right|/D_{qua}$$

(19) $$\left|V_{pre}-V_{Tru}\right|\leq D_{qua}$$

(20) $$R=-(\left|V_{pre}-V_{Tru}\right|-D_{qua})/{(D_{far}-D}_{qua}/4)$$

$V_{pre}$ is the predicted change of the agent’s estimated action combined with the weighted calculation. After defining the relationship between the actual change and the other two localization changes, it is compared with $V_{pre}$ to determine the reward value.

In another case, if it does not conform to Equation (17), which represents the situation that $V_{Tru}$ is close to $V_{ORB}$ or $V_{PLICP}$. It can also be recognized as when $V_{Tru}$ is extremely close to the variation of one of the localization algorithms. When $V_{Tru}$ is already close to the value predicted by the algorithm, the appropriate weight adjustment can achieve better results than the half-weight design. The pursuit of the goal is to predict the value that is a perfect fit for the actual amount of change. Therefore, if Equation (21) is satisfied, which represents that the spacing between $V_{pre}$ and $V_{Tru}$ is better than the effect of each half-weight design, then a positive reward value is given, and the output reward value *R* can be expressed as Equation (22). If Equation (21) is not satisfied, it means that the effect of this prediction is worse than the result of the adjustment of each half of the weights, and a negative reward value is given, resulting in the designed reward value *R* as in Equation (23).

(21) $$\left|V_{pre}-V_{Tru}\right|\leq {(D_{far}-D}_{max}/2)$$

(22) $$R=1-\left|V_{pre}-V_{Tru}\right|/{(D}_{max}/2-D_{clo})$$

(23) $$R=\left(-\left|V_{pre}-V_{Tru}\right|-{(D}_{max}/2-D_{clo})\right)/(D_{max}/2)$$

#### 2.3.2. Outside the Interval of Variation

This situation applies when the actual amount of change exceeds the range of the estimated amount of change of the two localization algorithms. That is, the actual amount of change can be greater or less than the amount of change in the positioning between ORBSLAM2 and PLICP at the same time, which conforms to one of the conditions in Equation (24) or (25).

(24) $$V_{Tru}\geq \;V_{ORB}\;and\;V_{Tru}{\geq V}_{PLICP}$$

(25) $$V_{Tru}\leq V_{ORB}\;and\;V_{Tru}\leq V_{PLICP}$$

For the design of the reward value, this paper first calculated the absolute error value $D_{max}$ between ORBSLAM2 and PLICP and took one-half of $D_{hal}$ as the criterion for judgment. After the adjustment of the weights, the goal of predicting the localization variation was to approximate the value of the localization algorithm with $V_{Tru}$ more closely, as shown in Equation (26). The prediction goal of this design was to match the actual change as much as possible. It is expected to get better than the adjustment effect of each half-weight. Therefore, the design concept of the reward value was to go through the absolute value of the difference between the weighted predicted variability $V_{pre}$ and the error in the two localization algorithms as a criterion. It is mainly according to the following algorithm to complete the reward value of the region, which is described as follows:

Assuming that Equation (26) is satisfied, it means that this prediction is very close to the actual position of the mobile robot. So, a positive reward value of *R* is given, as shown in Equation (27). If Equation (26) is not satisfied, it means that the result of this prediction is worse than the effect of half-weights. So, a negative reward value of *R* is given, as shown in Equation (28).

(26) $$\left(\left|V_{pre}-V_{Tru}\right|-D_{clo}\right)<(D_{max}/2)$$

(27) $$R=\left(\left|V_{pre}-V_{Tru}\right|-D_{clo}\right)/{(D}_{max}/2)$$

(28) $$R=-\left(\left|V_{pre}-V_{Tru}\right|-D_{clo}-{(D}_{max}/2\right)/{(D}_{max}/2)$$

**Figure 17:**
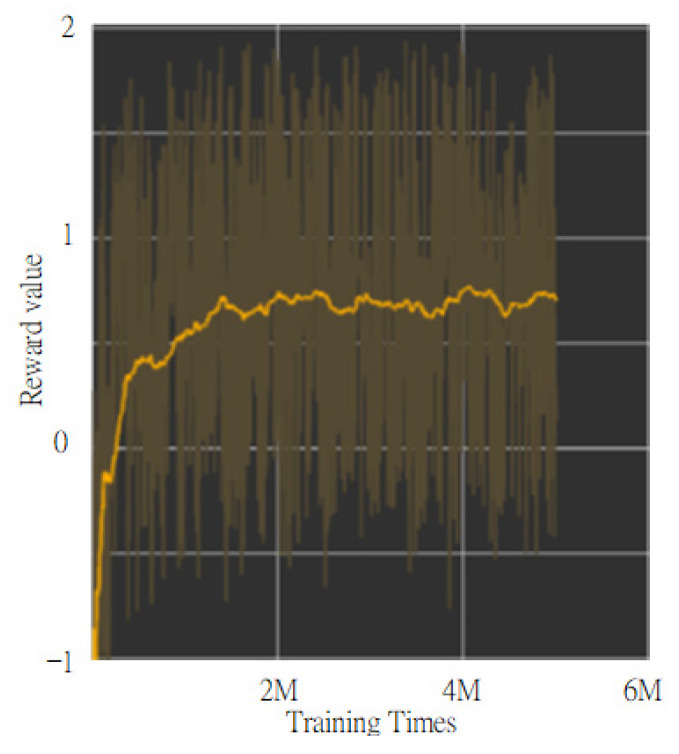
Result of reward function.

The overall experimental results through the designed reward function are shown in Figure 17. In this study, the reward function was proposed in such a way that the *x*-axis and *y*-axis were trained at the same time. It is illustrated that the total reward value obtained at each step of the training time would be between −2 and 2. The yellow line is the curve of the actual reward value after smoothing, while the dark yellow line is the curve of the actual reward value. It can be clearly seen that after about 2 million steps of training, the total reward value obtained by the reward function gradually approached about 0.72 or so. As long as the reward value of each step of the reward function is greater than 0, it means that the weights of the algorithms trained by this deep reinforcement learning are able to increase their performance. Ultimately, the trained network model with DRL can be accomplished with fast convergence and strong adaptability.

## 3. Experiment Result in Mobile Robot Localization

To verify the accuracy of the localization method, the actual positions and postures of the mobile robots were obtained from the simulation environment. All the required data in the simulation environment was made into a dataset to ensure that the same data were used in each experiment. The ORBSLAM2, PLICP, the combination of half-weights, and the deep learning weight adjustment method of PPO proposed in this paper were used in the dataset for the comparison of the accuracies. In order to demonstrate that our combined approach can effectively compensate for the shortcomings of the two localization algorithms, the PLICP algorithm was degraded in environments with low-point cloud continuum smoothness. The ORBSLAM2 localization effect was even worse than that of the LiDAR type. Therefore, the maps of the two environments were created in the Gazebo simulation environment for localization tests, which are classified into (1) simulation scenario 1 and (2) simulation scenario 2.

### 3.1. Simulation Scenes

**Figure 18:**
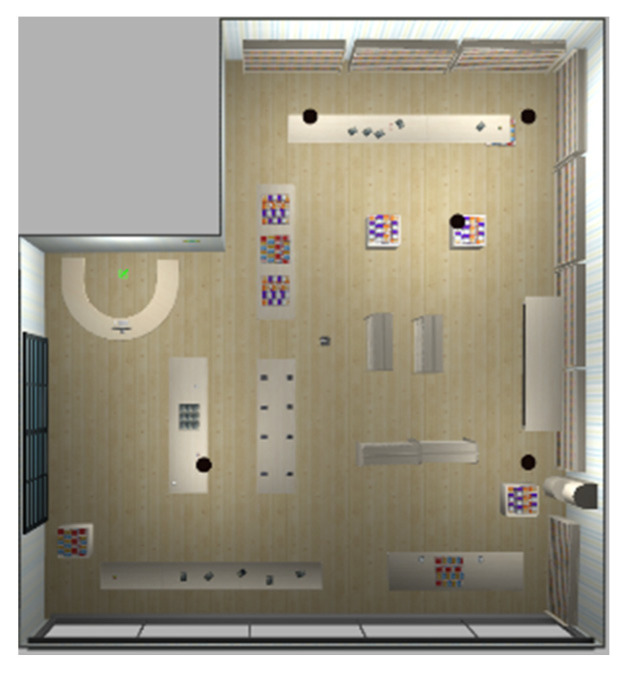
Simulation Scene 1.

**Figure 19:**
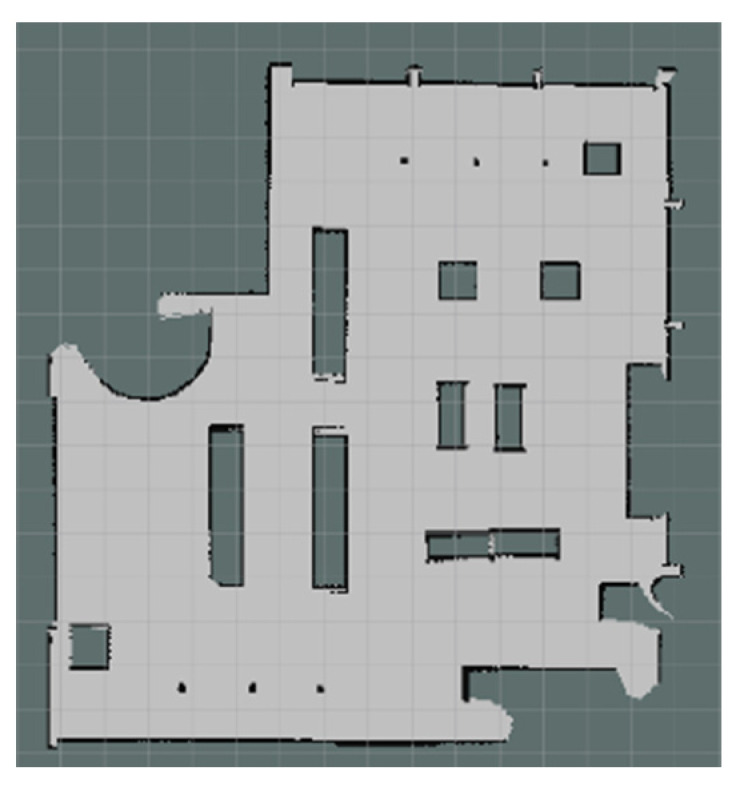
Occupancy grid mapping for Simulation Scene 1.

**Figure 20:**
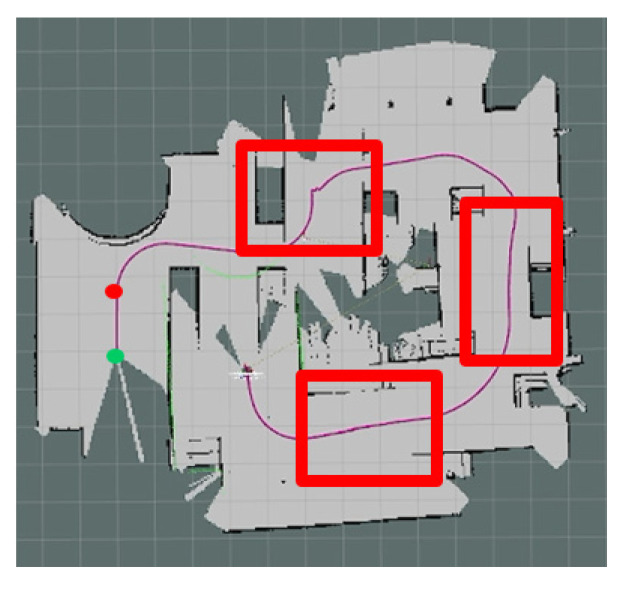
Minimal error map for original PLICP area. (The green point is the start point of the mobile robot, the red point is the end point of the mobile robot, and the red box is the main area that causes the error).

**Figure 21:**
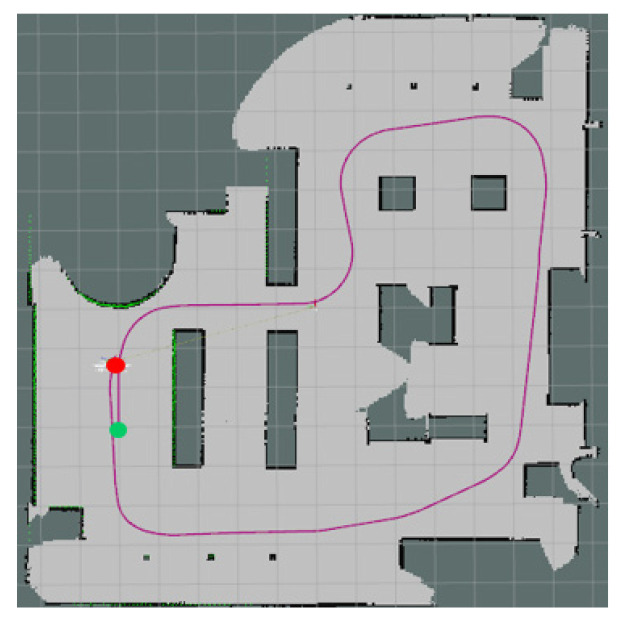
Minimal error map of PLICP area after improvement. (The green point is the start point of the mobile robot, the red point is the end point of the mobile robot).

The experiments in Simulation Scene 1 modified the graphs of the bookstore [36] simulation environment in Amazon Web Services (AWS). Its main purpose is to create an environment with a high degree of point cloud correction and continuous smoothing. Most of the objects in this scene were relatively neat and smooth to the point cloud scanned by the LiDAR. The completed diagram of simulation scene 1 is shown in Figure 18, and the occupancy grid map based on the LiDAR point cloud created by the method in this study is shown in Figure 19. The occupancy grid map shows that most of the objects on the map have square edges. The PLICP algorithm is prone to reaching the threshold for termination of the algorithm early due to the environmental factor of square edges, which resulted in the error of region minimization, as shown in Figure 20. The green point is the start point of the mobile robot, the red point is the end point of the mobile robot, and the red box is the main area that causes the error. It can be seen that the final position of the mobile robot had a small error with the endpoint. In the experimental part, the initial original PLICP obtained the best mean square error of 12.14 cm, and the improved PLICP experimental result obtained a better mean square error of 10.93 cm, as shown in Figure 21.

Since real-world environments are often suboptimal, we have altered the AWS small-warehouse [37] simulation environment. We hope to create an environment similar to real-world environments such as factories, convenience stores, etc. where the scene is more complex, i.e., a scene with a lower point cloud continuum smoothness.

**Figure 22:**
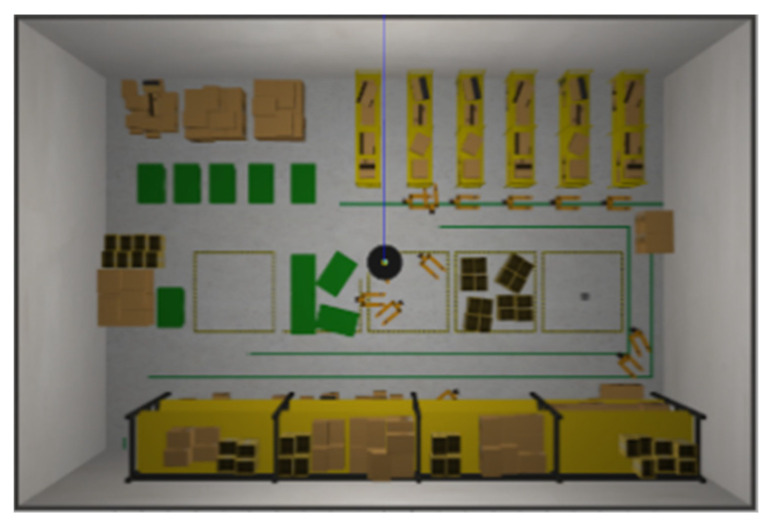
Simulation Scene 2.

**Figure 23:**
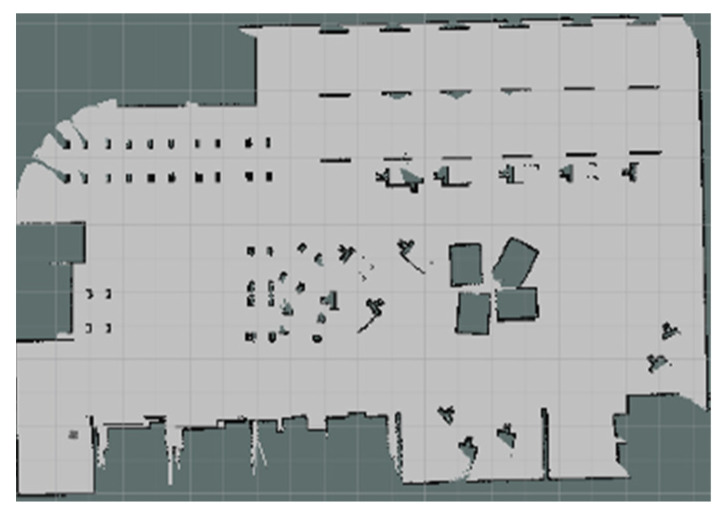
Occupancy grid mapping for Simulation Scene 2.

**Figure 24:**
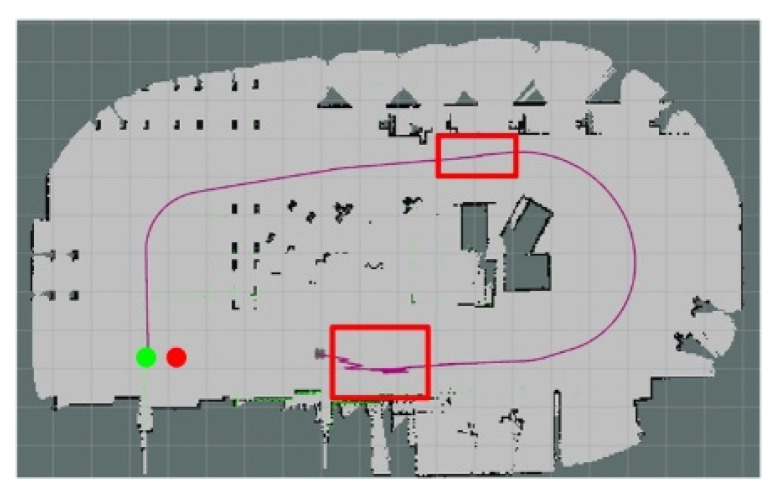
Minimal error map for original PLICP area in Scene 2. (The green point is the start point of the mobile robot, the red point is the end point of the mobile robot, and the red box is the main area that causes the error).

**Figure 25:**
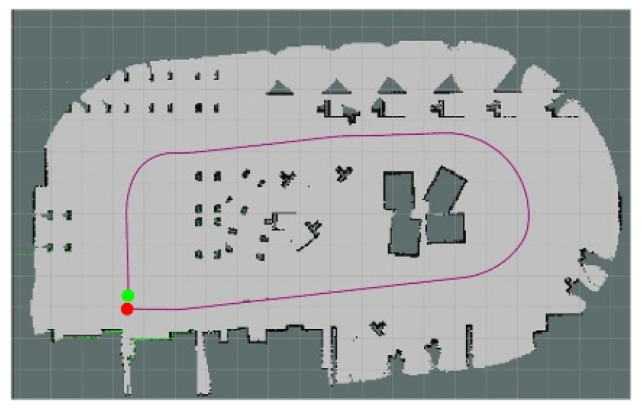
Minimal error map of PLICP area after improvement in Scene 2. (The green point is the start point of the mobile robot, the red point is the end point of the mobile robot).

The actual context diagram of Simulation Scene 2 is shown in Figure 22. Most of the objects were relatively heterogeneous to the point cloud of the LiDAR. The occupancy grid map based on the LiDAR point cloud is shown in Figure 23. Figure 24 shows the occupancy grid map generated based on the LiDAR point cloud. The green point is the start point of the mobile robot, the red point is the end point of the mobile robot, and the red box is the main area causing the error. This original Scene 2 map also resulted in a small error in region minimization. Figure 25 shows the improved map by the PLICP algorithm. This result shows that the PLICP algorithm can actually improve the error value significantly. In this experiment, the original PLICP map obtained the best mean square error of 2.18 cm, and the improved PLICP experimental result obtained a better mean square error of 0.87 cm. This can be seen on the right-hand side of the occupancy grid map, where most of the objects on the map were rather cluttered and had a similar environment to a factory.

### 3.2. Simulation Test

In this paper, 10 consecutive tests were carried out in simulation environment 1, and the accuracy comparison table obtained by getting the mean square error (MSE) with the actual localization of the mobile robot is shown in Table 2. Experimental results from Simulation Environment 1 showed that 80% of our method was more accurate than the method with half of the weights. Also, 50% of the results were better than the localization of the traditional ORBLSAM2 and PLICP algorithms alone at the same time.

A comparison of the accuracy obtained from 10 consecutive tests performed in Simulation Environment 2 with the MSE of the actual localization of the mobile robot is shown in Table 3. The experimental results from Simulation Environment 2 showed that 90% of our method was more accurate than the one with half of the weights, and 70% of the results were better than the localization of both the traditional ORBLSAM2 and PLICP algorithms. Further, there was no case in which the results were worse than those of the traditional ORBSLAM2 and the PLICP algorithms at the same time. The positioning error is also minimized to an average of 10 times.

As shown in the above experimental data, the method in this paper can achieve more accurate localization results when tested in a flat environment with a high degree of smoothness and a complex environment with a low degree of smoothness in the point cloud continuum. It has been proven that the proposed method can achieve more accurate and robust localization results.

## 4. Conclusions

In this study, we proposed a multi-fusion that outperforms the traditional LiDAR point-to-line iterative closest point (PLICP) and Camera ORBSLAM2 algorithms for SLAM localizations, which are widely used at present. These two localization algorithms were combined with the trained deep reinforcement learning networks and MMAE to concurrently complete the sensor fusion for approaching the desired targets. The experimental results showed that the proposed method can achieve the localization goal more accurately in different simulated environments. The main contributions of the research presented in this study are listed as follows:

(1) The localization algorithm proposed in this paper includes the concept of fault detection by setting a threshold value through the discrepancy of the residual values and also referring to the speed of the current mobile robot. It can detect the possible failure conditions of different sensors in real time and then calculate the confidence value of the current prediction of the two localization algorithms through hypothesis checking after detecting the failure. At the same time, it intervenes to adjust the weight of the current changes in the positioning of the sensor. Experimental results show that its proposed method can effectively detect anomalous values and immediately rule out anomalous localization. Thus, the robustness of the sensory fusion localization method for multi-model adaptive estimation is improved.
(2) In this paper, deep reinforcement learning is used to complete the weight adjustment, and the input two-sensor localization algorithm is trained with the predicted variations and residual values. The trained system accepts inputs related to the localization algorithm and predicts the weights to be adjusted for different environmental information. The results show that a more robust weight adjustment mechanism can be established and achieve superior positioning accuracy in different environments.
(3) Since this PLICP method can easily lead to the problem of minimal area under the environment of high-point cloud continuous smoothness, this paper used odometry information to improve the design of PLICP in calculating the displacement change and rotation change as the initial values of the first conversion relationship. This can effectively solve the problem of the minimal area by reducing the number of iterations and computing power. This method can also increase the information in the odometry, which can enhance the correlation between the sensor fusion.

In the future, this study can be extended to improve localization accuracy with other appropriate concepts, such as collaborative localization with different sources [38] or other types of artificial intelligence indoor localization methods through wireless sensors [39], to achieve better results.

## Figures and Tables

**Table 1:** Hypothesis testing decision table.

Table of Error Types		$\mathrm{Null}\;\mathrm{Hypothesis}\;(H_{0}$)	
		True	False
Decision about Null Hypothesis ($H_{0}$)	Accept	Correct Decision	Type II Error
	Reject	Type I Error	Correct Decision

**Table 2:** Precision comparison table for simulation environment 1. (Bold and underline parts are the best result in each test).

Times	ORBSLAM2 [30]	PLICP [29]	Half Weight	Ours
1	0.5319	0.2914	0.2013	**0.1907**
2	0.4274	**0.0337**	0.1061	0.0898
3	0.5503	0.3183	0.1495	**0.1155**
4	0.3403	**0.0929**	0.1215	0.1250
5	0.4524	**0.0861**	0.1282	0.0975
6	0.4037	**0.1001**	0.1697	0.1784
7	1.0574	0.2076	0.2355	**0.1595**
8	0.3671	0.1263	0.1310	**0.1137**
9	0.2511	0.2744	0.1343	**0.1279**
10	0.4908	**0.0487**	0.1738	0.1529

**Table 3:** Precision comparison table for simulation environment 2. (Bold and underline parts are the best result in each test).

Times	ORBSLAM2 [31]	PLICP [29]	Half Weight	Ours
1	0.4723	0.4089	0.3818	**0.3795**
2	1.4706	0.3065	0.3075	**0.2403**
3	0.6790	0.4268	0.2963	**0.2849**
4	0.4001	0.5688	0.4135	**0.3681**
5	0.7822	0.4013	**0.3086**	0.4308
6	1.1127	0.9989	1.0556	**0.9582**
7	0.6071	0.2317	0.1992	**0.1807**
8	0.3400	0.3325	0.2515	**0.2136**
9	2.5652	**0.2875**	0.7886	0.7007
10	0.2584	**0.0692**	0.1267	0.1109

## Data Availability

Data are contained within the article.
